# Comparison of detection methods and follow-up study on the tyrosine kinase inhibitors therapy in non-small cell lung cancer patients with ROS1 fusion rearrangement

**DOI:** 10.1186/s12885-016-2582-9

**Published:** 2016-08-04

**Authors:** Jieyu Wu, Yunen Lin, Xinming He, Haihong Yang, Ping He, Xinge Fu, Guangqiu Li, Xia Gu

**Affiliations:** 1 Department of Pathology, the First Affiliated Hospital of Guangzhou Medical University, No. 151, Yanjiangxi Road, Guangzhou, 510120 China; 2 Department of Cardiothoracic Surgery, the First Affiliated Hospital of Guangzhou Medical University, Guangzhou, China

**Keywords:** ROS1, Immunohistochemistry, Fluorescent *in situ* hybridization, Quantitative real-time polymerase chain reaction, Non-small cell lung cancer, Tyrosine kinase inhibitors

## Abstract

**Background:**

The screening of ROS proto-oncogene 1, receptor tyrosine kinase(ROS1) fusion rearrangement might be potentially beneficial for an effective therapy against non-small cell lung cancer (NSCLC). However, the three main ROS1 rearrangement detection methods have limitations, and no routine protocol for the detection of ROS1 rearrangement in NSCLC is available. In this study, our aims were to compare immunohistochemistry (IHC), fluorescent *in situ* hybridization (FISH) and quantitative real-time polymerase chain reaction (qRT-PCR) in their ability to detect ROS1 rearrangement in NSCLC, and discuss the clinical characteristics and histopathology of the patients with ROS1 rearrangement. Moreover, the effects of tyrosine kinase inhibitors (TKIs) therapy on the patients with ROS1 rearrangement and advanced stage disease (III b–IV) were investigated.

**Methods:**

Patients with a previously diagnosed NSCLC were recruited in this study from November 2013 to October 2015. IHC was performed using the D4D6 monoclonal antibody (mAb) in an automatic IHC instrument, while FISH and qRT-PCR were carried out to confirm the IHC results. FISH and qRT-PCR positive cases underwent direct sequencing. After detection, patients with advanced ROS1 rearranged NSCLC had received TKI therapy.

**Results:**

Two hundred and thirty-eight patients were included in this study. ROS1 rearrangement was detected in 10 patients. The concordant rate of FISH and qRT-PCR results was 100 %, while in the FISH and IHC results high congruence was present when IHC showed a diffusely (≥60 % tumor cells) 2–3+ cytoplasmic reactivity pattern. Patients harboring ROS1 rearrangement were mostly young (8/10), females (7/10) and non-smokers (7/10) with adenocarcinoma (10/10) and acinar pattern. Most of their tumor were in intermediate grade (6/8). Among these 10 patients, three of them in stage IV with ROS1 rearrangement gained benefits from ROS1 TKI therapy.

**Conclusions:**

IHC, FISH and qRT-PCR can reliably detect ROS1 rearrangement in NSCLC, while IHC can be used as a preliminary screening tool. These results supported the efficacy of ROS1 TKI therapy in treating advanced NSCLC patients with ROS1 rearrangement.

**Electronic supplementary material:**

The online version of this article (doi:10.1186/s12885-016-2582-9) contains supplementary material, which is available to authorized users.

## Background

Mutations in receptor tyrosine kinases (RTKs) genes have been identified as the main cause of many carcinomas development, since they can lead to proliferation and transformation of cancer cells [1]. In recent years, ROS proto-oncogene 1, receptor tyrosine kinase (ROS1), a gene located on 6q22, which transcripts the protein that belongs to the subfamily of tyrosine kinase insulin receptor, has been recognized as a driver of non-small cell lung cancer (NSCLC) [2] since it can fuse with other genes (e.g. CD74, SLC34A2, FIG, TPM3, SDC4, EZR, LRIG3, CCDC6, and KDELR2 [3, 4]) and consequently activate the downstream growth and survival signaling pathways [3–7]. In most cases, ROS1 fusion rearrangement is exclusive to other RTK aberrance, such as the anaplastic lymphoma receptor tyrosine kinase (ALK) rearrangement, epidermal growth factor receptor (EGFR) mutations and Kirsten rat sarcoma viral oncogene homolog (KRAS) mutations [4]. Moreover, because of the homology between the ROS1 and ALK proteins [8, 9], patients with ROS1 rearrangement are sensitive to ALK tyrosine kinase inhibitors (TKIs). Therefore, despite the incidence of ROS1 rearrangements in NSCLC is low (1–2 %) [4, 10], screening ROS1 rearrangement could be potentially beneficial for NSCLC patients.

In the present work, fluorescent *in situ* hybridization (FISH), quantitative real-time polymerase chain reaction (qRT-PCR) and immunohistochemistry (IHC) have been used for ROS1 arrangement detection. All of these methods have advantages and limitations. FISH analysis can reveal the genes rearrangement status, but the procedure is inconvenient [11, 12], and it is not suitable for biopsies with insufficient numbers of tumor cells. qRT-PCR analysis can reveal fusion rearrangements by using specific primers and it has a high sensitivity. However, qRT-PCR cannot detect specimens with unknown fusion types [11, 12]. IHC is feasible in large scale screening, and the D4D6 rabbit monoclonal antibody (mAb) has been identified as effective and specific mAb for ROS1 rearrangement protein detection by several studies [3, 8, 11]. In addition, the costs to perform IHC are less compared with qRT-PCR or FISH. However, there is not an accurate cutoff value to define positive ROS1 protein expression using IHC, thus representing a limitation on using this method [11–14]. Therefore, the aim of this study was to compare these three analytical methods in their ability to detect ROS1 rearrangement in NSCLC, trying to set up a cutoff value for ROS1 IHC analysis. In addition, we investigated the efficacy of TKI therapy in treating advanced NSCLC patients with ROS1 rearrangement. The characteristics of NSCLC patients harboring ROS1 rearrangement were also discussed.

## Methods

### Patient selection

Patients admitted to the First Affiliated Hospital of Guangzhou Medical University were screened and recruited for this study from November 2013 to October 2015. Patients were selected upon (1) a previous identification of NSCLC with (2) a confirmed diagnosis by IHC of p63, CK5/6, NapsinA and TTF-1 protein expression [15]. A cohort of 238 NSCLC patients was included. Afterwards, all slides from the chosen cases were independently analyzed by two pathologists (X Gu & JY Wu) blinded to history and prior diagnoses. The histopathological classification was performed according to the 2015 WHO classification of lung tumors [15] and the International Association for the Study of Lung Cancer/American Thoracic Society/European Respiratory Society (IASLC/ATS/ERS) multidisciplinary classification [16]. Appropriate specimens with sufficient tissue (>100 tumor cells) were included.

After recruitment, the clinical information, including age, gender, smoking history, and tumor node metastasis (TNM 7th) staging were collected. In adenocarcinoma cases, the histological grading was performed by analyzing the single most predominant pattern in a case [15]. According to 2015 WHO histological grading of adenocarcinoma, the grading was divided into low, intermediate and high. Another grading score system that combined the most two predominant pattern in a case was also been used, which was worked out by Sica et al. [17]. The results of other genetic markers testing were also collected, such as ALK, EGFR and KRAS. Ventana IHC with D5F3 mAb and FISH with break-apart probe were used in ALK rearrangement detection. Amplification refractory mutation system polymerase chain reaction (ARMS-PCR) was used to detect EGFR and KRAS gene mutation. This study was approved by the Ethic Review Committee of the First Affiliated Hospital of Guangzhou Medical University.

### Immunohistochemistry (IHC)

All the specimens were formalin-fixed and paraffin embedded (FFPE). ROS1 IHC was performed on 4 μm slides and completed on a fully automated IHC instrument (BenchMark XT, Roche, Switzerland). D4D6 rabbit mAb (Cell Signaling Technology, Danvers, MA) diluted in 1:200 was used as primary antibody. Detection was using UltraView Universal DAB detection Kit (Roche, Switzerland). IHC was scored using the following score scheme: 0, no staining of tumor cells; 1+, tumor cells with faint cytoplasmic reactivity without any background staining; 2+, tumor cells with moderate cytoplasmic reactivity; and 3+, tumor cells with strong granular cytoplasmic reactivity [11]. When several intensity levels present in a case, it was scored according to the intensity of major tumor cells. The extent of IHC staining was also analyzed by estimating the staining percentage of tumor cells [8]. Moreover, H-score method was used and calculated using the following equation: H-score = ∑[intensity (0, 1, 2, 3) × extent of each staining intensity(%)], with a scoring range from 0 to 300 [14]. Previous lung specimens with ROS1 rearrangements confirmed by FISH and a 3+ staining score, have been used as positive control. IHC was analyzed independently by two pathologists (X Gu & JY Wu), and disagreements were discussed after the analysis. A third pathologist (XG Fu) was invited as the reviewer when an agreement could not be reached. The above results were blinded for the qRT-PCR results.

### Quantitative real-time polymerase chain reaction (qRT-PCR)

Total RNA was isolated from FFPE tissue sections (6 μm slides) using the FFPE RNA Kit (Amoy Diagnostics Co., Ltd, Xiamen, China). RNA concentration was measured using a spectrophotometer (Nanodrop 2000c, Thermo-Scientific, Wilmington, US) and reverse transcription was performed to generate complementary DNA (cDNA). The cDNA was used for multiple RT-PCRs that were carried out in an Mx3000p real-time PCR system (Agilent Technologies, California, US) using the ROS1 Gene Fusion Detection Kit (Amoy Diagnostics Co., Ltd, Xiamen, China). The positive and negative reference samples were also used. The PCR procedure was the following: One cycle at 95 °C for 5 min; 15 denaturation cycles at 95 °C for 25 s, annealing at 64 °C for 20 s and elongation at 72 °C for 20 s; 31 cycles at 93 °C for 25 s, 60 °C for 35 s (data collection) and 72 °C for 20 s. The quantification is determined by the fusion fluorescence signals and the assay with a Ct value < 30 cycles was considered as positive. These results were blinded for the IHC and FISH results.

### Tissue microarray (TMA) and fluorescent *in situ* hybridization (FISH)

IHC positive staining areas were evaluated and selected from the slides by a pathologist (JY Wu) to avoid tumor heterogeneity and the tissue microarray (TMA) was performed from the FFPE samples. Two areas of 2 mm diameter were removed from each sample block using a stainless steel stylet (Xinsen, Jieli Biomedicine Co., Ltd, Guangzhou, China). Serial 4 μm TMAs sections were used for FISH detection using 6q22 ROS1 Break Apart FISH Probe RUO Kit (Abbott Molecular Inc, IL, USA). The protocol and interpretation of FISH were the following: TMA slides were submerged in xylene and decreasing gradient of ethanol for deparaffinization and hydration, respectively. Next, they were subjected to a heat-treatment in boiled water (100 °C, 30 min) and digestion using proteinase K (37 °C, 5 min). They were washed in 2 × SSC solution and dehydrated by increasing gradient of ethanol (70 %, 85 % and 100 %) for 3–5 min. After air drying, the probe was added to the target specimens, and coverslips were placed. The slides were placed in the hybridization machine (ThermoBrite, Abbott Molecular Inc, IL, US) and hybridization was performed as follows: denaturation at 75 °C for 8 min and hybridization at 42 °C for 16 h. Next, the slides were washed in 2 × SSC and NP40 solution at 42 °C for 5 min and immersed in 70 % ethanol for 5 min. DAPI 15 μl was applied to counterstain. Analysis was performed in the dark using the fluorescence microscopy (Nikon 80i, Japan). The data analysis was the following: >15 % tumor cells showing split signals (“red” and “green” split signals) or isolated 3′ signals (single “green” signals) belonged to the ROS1 fusion rearrangement. These results were blinded for the qRT-PCR results.

### Direct sequencing

The cDNA of FISH and qRT-PCR positive cases were sent to Amoy Diagnostics Co., Ltd for direct sequencing. The results of the sequencing were compared using the Basic Local Alignment Search Tool (BLAST).

### Follow-up visits

After ROS1 rearrangement detection using IHC, FISH and qRT-PCR, the patients harboring ROS1 rearrangement in advanced stages (III b–IV) of disease were selected for TKI therapy. In order to track the efficacy of the therapy, information such as patient’s syndromes, vital signs and CT images were collected every two months. The efficacy was evaluated using RECIST guideline 1.1 [18]. The materials of patients were authorized by the recruited patients and (or) their family members.

### Statistical analysis

Pearson’s *χ*^2^ and Fisher’s exact test were used to assess the relationship between ROS1 rearrangement, clinical characteristics and clinicopathological patterns. The Kappa value was calculated to assess the concordant rate of FISH and IHC in detecting ROS1 rearrangement. The analyses were carried out using the Statistical Package for the Social Sciences (SPSS) version 13.0 (SPSS, Inc., Chicago, IL, US), and *P* values less than 0.05 were considered statistically significant.

## Result

### Characteristics of the recruited cases

Two hundred and thirty-eight cases were recruited, of which 215 were surgical resection cases and 23 were needle biopsy cases. The clinical characteristics and histopathology of the patients are shown in Table 1. The median age was 61 years old (range from 27 to 85 years old), and 107 were females and 131 were males. Most of the included cases were in the early stages (114/238, 47.9 %) of the disease, while 48 (48/238, 20.2 %) cases were in the advanced stages (III b–IV). Total 181 resected adenocarcinoma cases were performed histological grading, they mostly obtained score 5 in Sica staging (69/181, 38.1 %) and classified as intermediate grade in the WHO grading (126/181, 69.6 %). Details of the grading were showed in a supplementary table [see Additional file 1: Table S1]. However, there was no statistical difference between ROS1 rearrangement and non-rearrangement cases in clinical characteristics. Two hundred and twenty-eight patients underwent ALK rearrangement detection, 163 and 153 patients underwent EGFR and KRAS mutation detection, respectively. Among these cases, 12 cases (12/228, 5.3 %) were harboring ALK rearrangement, 87 cases (87/163, 53.4 %) and 13 cases (13/153, 8.50 %) were harboring EGFR and KRAS mutation, respectively.

**Table 1:** Characteristic of included cases

Characteristic		Total	Surgery resection	Biopsy	ROS1 rearrangement	ROS1 non–rearrangement	*P.*
No.		238	215	23	10	228	
Age							0.114^a^
	>61	109	101	8	2	107	
	≤61	129	114	15	8	121	
Gender							0.118^a^
	Male	131	118	13	3	128	
	Female	107	97	10	7	100	
Smoking history							0.792^a^
	Smoker	51	48	3	3	48	
	Non–smoker	158	140	18	6	152	
	Previous smoker	29	27	2	1	28	
Histopathology							1.000^a^
	ADC	216 (8)^b^	195 (6)^b^	21 (2)^b^	10	206 (8)^b^	
	SCC	11	9	2	0	11	
	ASC	2	2	–	0	2	
	LCLC	9 (1) ^b^	9 (1) ^b^	–	0	9 (1) ^b^	
Adenocarcinoma subtypes (predominant pattern)^c^							0.207^a^
	Lepidic	17	17	–	2	15	
	Acinar	105	105	–	4	101	
	Papillary	33	33	–	3	30	
	Micropapillary	16	16	–	2	14	
	Solid	20	20	–	0	20	
	Invasive mucinous adenocarcinoma	8	8	–	1	7	
	Fetal	1	1		0	1	
TMN stage^d^							0.175^a^
	I	114	114	0	6	108	
	II	30	29	1	1	29	
	III	55(IIIb:10)	53	2	0	55	
	IV	38	18	20	3	35	
Sica grading^e^							0.871^a^
	Score 2	6	6	-	0	6	
	Score 3	48	48	-	3	45	
	Score 4	48	48	-	1	47	
	Score 5	69	69	-	4	65	
	Score 6	10	10	-	0	10	
WHO grading^e^							0.597^a^
	Low grade	17	17	-	1	16	
	Intermediate grade	126	126	-	5	121	
	High grade	38	38	-	2	36	
Another gene status							–
	ALK	228 (12)^f^	206 (11)^f^	22 (1)^f^	0	228	
	EGFR	163 (87)^f^	141 (78)^f^	22 (9)^f^	0	163	
	exon 19 deletion	37	33	4	0	37	
	L858R	48^g^	44^g^	4	0	48	
	L861Q	1	1	0	0	1	
	exon 20 S768I	2^g^	1^g^	1	0	2	
	KRAS	153 (13)^f^	136 (11)^f^	17 (2)^f^	0	153	
	Gly12Asp	3	3	0	0	3	
	Gly12Cys	4	4	0	0	4	
	Gly12Val	4	4	0	0	4	
	Gly12Ala	1	0	1	0	1	
	Gly12Ser	1	0	1	0	1	

Abbreviations: *ADC* Adenocarcinoma, *SCC* Squamous cell carcinoma, *ASC* Adenosquamous carcinoma, *LCLC* Large–cell lung carcinoma, *ALK* anaplastic lymphoma receptor tyrosine kinase, *EGFR* epidermal growth factor receptor, *KRAS* Kirsten rat sarcoma viral oncogene homolog

^a^Fisher exact test

^b^Metastasis cases

^c^Total 190 resected adenocarcinoma with 200 predominant patterns were discussed. Some cases were including more than one predominant patterns

^d^A case was diagnosed as stage 0

^e^Total 181 resected adenocarcinoma have been analyzed, excluding variant subtypes

^f^The cases with ALK rearrangement, EGFR mutation or KRAS mutation

^g^There was a case harboring both exon 21 L858R and exon 20 S768I mutation

### Comparison of IHC, FISH and qRT-PCR in ROS1 rearrangement detection

All the recruited patients underwent FISH and IHC detection of ROS1 rearrangement, and qRT-PCR analysis was applied in 159 cases. A total of 10 cases were confirmed as ROS1 rearrangement positive by FISH (10/238, 4.2 %; Table 2). Six of them underwent qRT-PCR detection, which confirmed the presence of ROS1 rearrangement. All qRT-PCR negative cases were also confirmed as ROS1 rearrangement negative by FISH.

**Table 2:** The characteristics of patients with ROS1 rearrangement

Cases No.	Smoking^a^	Staging/Grading^b^	IHC result^c^	H-score	FISH result	Direct sequencing	Histopathology predominant pattern	Staining Pattern	Another gene aberrance^d^
1	N	II B/ Score 5/Grade 2	3+/90 %	260	Fusion positive	SLC34A2-E4; ROS1-E32/SLC34A2-E4; ROS1-E34	Acinar	Cytoplasmic; membrane	ALK\EGFR\KRAS(−)
2	N	I A/Score 3/Grade 2/	2+/65 %	150	Fusion positive	CD74-E6; ROS1-E34	Acinar	Cytoplasmic; focal granular	ALK\EGFR\KRAS(−)
3	P	IV/−/−	2+/70 %	160	Fusion positive	CD74-E6; ROS1-E34	Invasive mucinous adenocarcinoma	Mucinous staining	ALK\EGFR\KRAS(−)
4	N	I A/Score 5/Grade 2	2+/90 %	200	Fusion positive	TPM3-E8; ROS1-E35	Papillary and acinar	Cytoplasmic; focal granular	ALK\EGFR\KRAS(−)
5	N	I A/Socre 5/Grade 3	2+/90 %	200	Fusion positive	SLC34A2-E14del; ROS1-E32/SLC34A2-E14del; ROS1-E34	Papillary and micropapillary	Cytoplasmic; focal granular	ALK\EGFR\KRAS(−)
6	N	IV/ Socre4/Grade 2	3+/85 %	250	Fusion positive	CD74-E6; ROS-E34	Acinar	Cytoplasmic; granular	ALK\EGFR\KRAS(−)
7^e^	N	IV/−/−	2+/70 %	160	Fusion positive	–	Invasive adenocarcinoma with acinar pattern	Cytoplasmic; focal granular	ALK\EGFR\KRAS(−)
8	S	I A/Score 5/Grade 3	3+/90 %	250	Fusion positive	–	Papillary and micropapillary	Cytoplasmic; focal granular	ALK\EGFR\KRAS(−)
9	S	I B/Socre 3/Grade 2	2+/80 %	180	Fusion positive	–	Acinar and lepidic	Cytoplasmic; focal granular	ALK\EGFR\KRAS(−)
10	N	I B/Score 3/Grade 1	2+/80 %	180	Fusion positive	–	Lepidic	Cytoplasmic; focal granular	ALK\EGFR\KRAS(−)

^a^ *N*, non-smoker; *S*, smoker; *P*, previous smoker

^b^TNM staging, SICA grading and WHO grading

^c^IHC results were containing intensity and extent scores

^d^ALK rearrangement, EGFR and KRAS mutation have been also investigated at the sme time. The cases harboring ROS1 rearrangement were exclusive to ALK rearrangement, EGFR and KRAS mutation

^e^cases 7 was biopsy sample

**Fig. 1:**
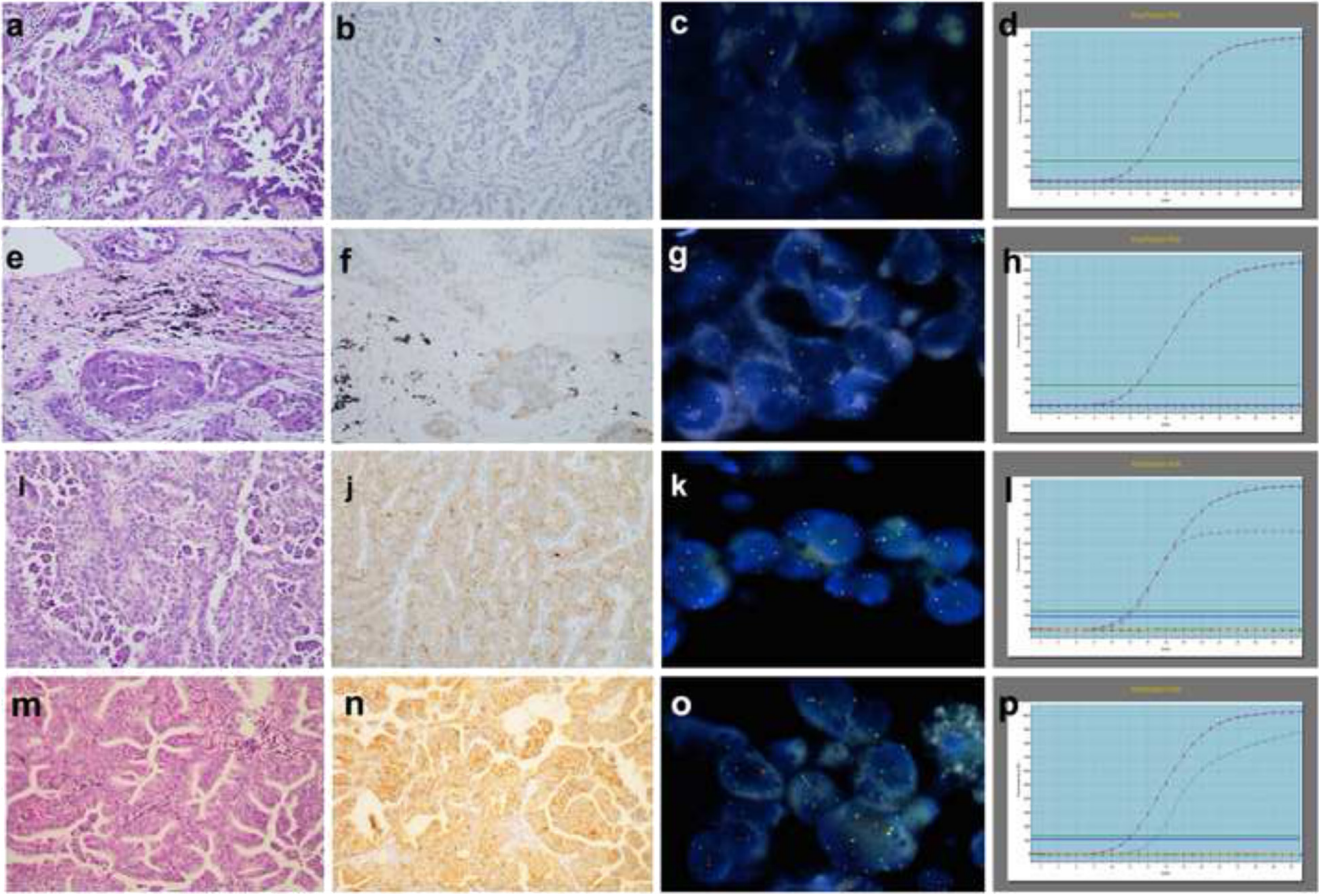
Comparison of IHC, FISH and qRT-PCR in ROS1 rearrangement and non-rearrangement cases (**a**–**d**). The H&E staining, IHC, FISH and qRT-PCR results of a non-rearrangement case. **a** The case presented acinar and papillary patterns in H&E staining (200×); **b** IHC showed no staining in tumor cells (200×); **c**, **d** it was confirmed by FISH and qRT-PCR as non-rearrangement, respectively; **e**–**h** A case with weak and focal ROS1 IHC staining. **e** The case presented acinar pattern in H&E staining (200×); **f** IHC showed weak to moderate focally staining in about 40 % tumor cells (200×); **g**, **h** it was also confirmed as non-rearrangement by FISH and qRT-PCR, respectively; **i**–**l** A case with diffusely moderate IHC staining. **i** The case presented papillary and micropapillary patterns in H&E staining (200×); **j** IHC showed moderate staining with cytoplasmic and focal granular patterns in about 80 % tumor cells (200×); **k** and **l** it was proved as ROS1-rearrangement by FISH and qRT-PCR, respectively; **m-p** A case with diffusely strong IHC staining. **m** The case presented papillary and micropapillary pattern in H&E staining (200×); **n** IHC showed diffusely strong staining with cytoplasmic, membrane and granular patterns in about 90 % tumor cells (200×); **o**, **p** it was also proved as ROS1 rearrangement by FISH and qRT-PCR

Forty-two cases showed cytoplasmic reactivity by IHC. Nevertheless, only ten cases with diffuse 2–3+ tumor cytoplasmic reactivity were confirmed as ROS1 rearrangement when FISH was set as the standard method. The staining was distributed in more than 60 % tumor cells (Table 2; Fig. 1j & n). A setting of 2+ in intensity, 60 % in extent, and an H-score of 150 as the cutoff value represented the optimal IHC settings to reach the highest sensitivity and specificity on ROS1 rearrangement detection (Table 3) [19]. A concordance between FISH and IHC was found when IHC showed moderate to strong cytoplasmic reactivity (2–3+) with diffuse (≥60 %) distribution or H-score≥ 150. (*P* < 0.01, Kappa value > 0.6; Table 4).

**Table 3:** H-score, intensity and extent of IHC

IHC	FISH+	FISH-	Sensitivity	Specificity
H-score				
≥50	10	18	100 %	92.1 %
≥100	10	5	100 %	97.8 %
≥150	10	0	100 %	100.0 %
≥200	5	0	50.0 %	100.0 %
≥250	3	0	30.0 %	100.0 %
Intensity				
1+	10	32	100 %	85.7 %
2+	10	1	100.0 %	99.6 %
3+	3	0	30.0 %	100 %
Extent				
≥10 %	10	29	100.0 %	87.28 %
≥20 %	10	25	100.0 %	89.04 %
≥30 %	10	20	100.0 %	91.23 %
≥40 %	10	15	100.0 %	93.42 %
≥50 %	10	14	100.0 %	93.86 %
≥60 %	10	9	100.0 %	96.05 %
≥70 %	9	9	90.0 %	96.05 %
≥80 %	7	4	70.0 %	98.25 %
≥90 %	4	1	40.0 %	99.56 %

**Table 4:** Comparison of FISH and IHC in ROS1 rearrangement detection

	FISH ROS1 fusion positive	FISH ROS1 fusion negative	*P*.	Kappa Value
IHC ROS1 2-3+	10	1	< 0.01^a^	0.950
IHC ROS1-, 1+	0	227		
IHC extent ≥ 60 %	10	9	< 0.01^a^	0.672
IHC extent < 60 %	0	219		
IHC H-score ≥ 150	10	0	< 0.01^a^	1.000
IHC H-score < 150	0	228		

^a^Fisher exact test

### Characteristics of the positive cases

Ten cases were identified as positive for ROS1 rearrangement. Most of the positive cases were female (Female: Male = 7:3) and non-smokers (7/10) with younger age (<61 year-old, 8/10). All the cases with ROS1 rearrangement were diagnosed as adenocarcinoma (Tables 1 and 2), and acinar pattern was the most predominant observed pattern. Eight cases could be performed histological grading, five of them got score 5 in Sica grading and 6 of them were classified as intermediate grade by WHO grading. Six of the rearrangement cases had been analyzed using direct sequencing (Table 2), revealing that CD74-E6 was the most common mutation type (3/6, 50 %). The images of direct sequencing are shown in an additional figure [see Additional file 2: Figure S1]. Most cases showed cytoplasmic and focal granular reactivity (7/10, 70 %; Table 2) [see Additional file 3: Figure S2, c & d]. No correlation was found between histopathology predominant patterns and IHC staining patterns (*P* = 0.645, Fisher exact test). All cases with ROS1 rearrangement were not carrying ALK, EGFR and KRAS gene aberrance.

The remaining 32 cases presented weak or focal IHC staining confirmed as ROS1 gene non-rearrangement or non-amplification by FISH. The staining patterns are shown in an additional figure [see Additional file 3: Figure S2]. All of them were diagnosed as adenocarcinoma. A case with an H-score of 90 and 2+ of intensity had been confirmed as ROS1 non-rearrangement by FISH. Its IHC staining was focal and represented approximately the 40 % of the tumor cells (Fig. 1f). Twenty-three of these cases underwent EGFR mutation detection, and 17 of them were confirmed as EGFR mutation (8 with exon 19 deletion and 9 with exon 21 L858R mutation). Thirty of them underwent ALK rearrangement detection, and two cases were confirmed as ALK rearrangement.

### Information related to the follow-up studies

Three patients (case 3, 6 and 7) (Table 2) belonging to the ten ROS1 rearrangement cases at the stage IV of their disease, received the therapy of crizotinib, a TKI approved by the Food and Drug Administration (FDA). The information related to these three patients is shown in Table 5. Details of these patients can be found in a supplementary material [see Additional file 4: Figure S3 (a-c)].

**Table 5:** The details of follow-up studies

Case no.	Surgery	Histopathology	Chemotherapy	ROS1 detection	CT imagine	TKI start time	Response	Reexamination CT imagine	Side effects
Patient 3	Video-assisted thoracic surgery (VATS) of the left upper lobe wedge resection	Invasive mucinous adenocarcinoma with pleural invasion	Pemetrexed, carboplatin	IHC, FISH and qRT-PCR had proved he as ROS1 rearrangement. Fusion type: CD74-E6	The largest lesion in his left thoracic wall was approximate in size to 40.81 × 12.70 mm^2^	Crizotinib 250 mg bid from December 2014	February 2015, the lesion decreased to 26.66 × 11.69 mm^2^ in size	October 2015, the largest lesion shrunk to 10.85 × 8.60 mm^2^ in size	Tiredness and constipation
Patient 6	Lower right lobe radical resection; Biopsy under CT guidance	Invasive adenocarcinoma with acinar predominant pattern	Pematrexed, nadaplatin and bevacizumab	IHC, FISH and qRT-PCR had proved she as ROS1 rearrangement. Fusion type: CD74-E6	The largest lesion was approximated in size to 36.25 × 36.25 mm^2^ on the pleura	Crizotinib 250 mg bid from April 2014	May 2014, the largest lesion decreased to 11.02 × 8.59 mm^2^ in size	October 2015, the largest lesion shrunk to 10.48 × 10.33 mm^2^ in size	Edema in lower limbs, vomiting and tiredness
Patient 7	Biopsy under CT guidance	Invasive adenocarcinoma with acinar pattern	–	IHC and FISH had proved she as ROS1 rearrangement	The largest lesion was approximate in size to 35.33 × 19.73 mm^2^	Crizotinib 250 mg bid from July 2014	September 2014, the lesion decreased to 26.97 × 15.12 mm^2^ in size	November 2015, her largest lesion shrunk to 16.25 × 5.65 mm^2^ in size	Tiredness

## Discussion

In the present study, three methods for ROS1 rearrangement detection have been compared. The results showed that D4D6 mAb IHC can be a reliable and feasible method for preliminary screening of ROS1 rearrangement in NSCLC, since it showed a high sensitivity and specificity. Nevertheless, the IHC cutoff value should be set at 2–3+ cytoplasmic reactivity with diffuse (≥60 % of the tumor cells) distribution or an H-score ≥150, which was similar to the conclusion of Yoshida’s study [14]. When we analyzed the pattern of IHC staining, we realized that the distribution of the cytoplasmic reactivity is one of the most important aspects in ROS1 IHC analysis. Indeed, a 2+ cytoplasmic reactivity intensity could be a false positive when the staining shows a focal distribution.

**Fig. 2:**
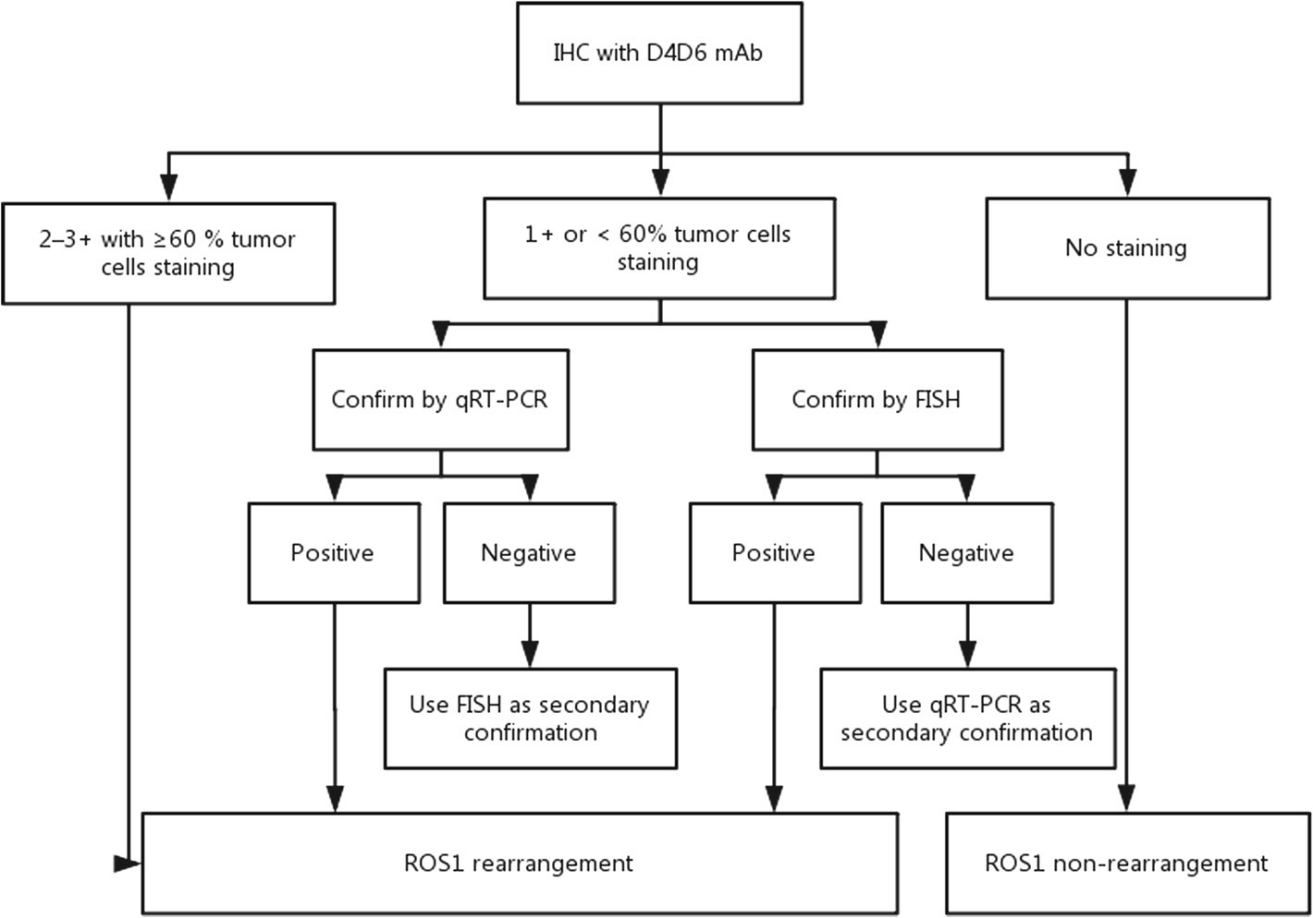
The routine protocol for ROS1 arrangements detection in NSCLC. IHC with D4D6 mAb can be preliminary screening tool for ROS1 rearrangement detection in NSCLC, and the IHC cutoff value should be set at 2–3+ cytoplasmic reactivity with diffuse (≥60 % of the tumor cells) distribution or an H-score ≥150; FISH and qRT-PCR should be used as the secondary confirmation in the cases with weak of focal IHC staining

In order to verify and confirm the weak or focal cytoplasmic reactivity, FISH and qRT-PCR should be used as secondary confirmation. In our study, the concordant rate between qRT-PCR and FISH was 100 %, indicating that qRT-PCR could be a reliable detection method for ROS1 rearrangement. In addition, some studies have indicated that FISH cannot clearly reveal the rearrangements on the same chromosome, such as GOPC (FIG)-ROS1 and EZR-ROS1 [4, 13]. Therefore, qRT-PCR can be used as a second confirmatory test for revealing these rearrangements. In contrast, the cases that resulted negative after qRT-PCR analysis should be confirmed by FISH since the primers of qRT-PCR do not contain unknown fusion partners [12]. Finally, we designed a protocol for the detection of ROS1 rearrangement shown in Fig. 2 that encompassed these considerations.

The clinical characteristics and pathological pattern of patients with ROS1 rearrangements were also discussed in this study. Even though there were no significant difference between ROS1 rearrangement and non-rearrangement patients, the cases with ROS1 rearrangement were mostly females at a younger ages and non-smokers with adenocarcinoma, which was similar to the results of some previous studies [20–22]. Most cases were classified as intermediate grade by WHO grading, however, five of them obtained score 5 in Sica grading, which indicated these cases containing high grade patterns as well. This result revealed that ROS1 rearrangement might become a prognosis biomarker of NSCLC. However, this finding should be confirmed in future study. Most ROS1 rearrangement cases presented cytoplasmic and focal granular staining pattern in the IHC staining, which was similar to the finding of some previous studies [14, 23]. However, the correlation between histopathology patterns and IHC staining patterns, as well as the correlation between fusion types and IHC reactivity patterns were not found due to the lack of ROS1 rearrangement cases. In addition, all the ROS1 rearrangement cases in our study were not carrying ALK rearrangement, as well as EGFR and KRAS mutations. Even though the overlapping phenomenon has been reported in some rare cases [8, 22], the result in our study indicated that general oncogene mutations not necessarily overlap in the same patient [4, 21].

Thirty-two cases with IHC weak or focal reactivity had been confirmed as ROS1 non-rearrangement. Although we had set the tissue with IHC strong reactivity as the positive control to avoid misunderstanding with the background staining, and chosen two areas with IHC reactivity in each FFPE block to decrease heterogeneity in establishing TMAs, these weak or focal staining might still be related to background staining or tissue heterogeneity. Nevertheless, among these cases, 17 were carrying EGFR mutation and 2 were harboring ALK rearrangement. To explain this phenomenon, Li et al. [24] investigated the expression of ROS1 mRNA in NSCLC, and found that the level of ROS1 mRNA increased either in ALK rearrangements or EGFR mutation specimens. However, the specific mechanism was unknown. We speculated that the weak or focal staining of ROS1 IHC may result from cross-talk mechanism of EGFR, ALK and ROS1 pathways, which is similar to the mechanism of EGFR mutation in NSCLC with MET proto-oncogene protein expression [25, 26]. However, EGFR mutation and ALK rearrangement detection was not possible on the remaining 9 and 2 cases, respectively. Thus, we cannot conclude that there was a correlation between weak or focal staining of ROS1 IHC and other genes aberrance.

After all the analyses, three patients with stage IV and ROS1 rearrangement underwent crizotinib therapy. All of them showed a partial response (PR) after 2 to 8 weeks, which was similar to some previous studies [8, 24]. In addition, even two patients had received chemotherapy before TKI therapy (patient 3 & 6), both of them had the same response to crizotinib as the other patient (patient 7), which indicated that crizotinib is also sensitive to the patients after chemotherapy. These three patients underwent crizotinib therapy for at least 11 months, reaching an average PR after 13.7 months. Since our patients were under a follow-up schedule, we could not estimate the progression-free survival (PFS). A large-scale study showed that the PFS of patients under crizotinib carrying ROS1 rearrangement was longer than the patients carrying ALK rearrangement undergoing the same therapy, suggesting the possible mechanism that imply crizotinib binding more tightly to ROS1 than to ALK [8].

Tiredness was the most common symptom during the therapy, and a patient (patient 6) also suffered from both lower limbs edema without cardiac dysfunction, which is one of the most common side effects observed in a previous study [8]. However, the correlation between TKI response and fusion partners had not been discussed because of the reduced ROS1 rearrangement cases to draw relevant conclusions. Because the FDA has recently approved crizotinib as a TKI to ROS1 rearrangement of NSCLC, we consider that the patients potentially harboring ROS1 rearrangement can be recruited by preliminary screening, therefore, more patients can receive TKI therapy, and the correlation between fusion types and TKI response or histopathology can be analyzed.

There are some limitations in our study. First of all, the qRT-PCR analysis was performed in only 159 cases. In order to analyze the concordance between qRT-PCR and FISH, all the recruited cases should be tested by qRT-PCR. However, some of the biopsied cases had insufficient amount of tissue to perform the qRT-PCR. Moreover, in order to clarify the relationship between fusion partners and TKIs response, and the correlation between fusion types and histopathology, more positive cases are needed. Finally, the relation between faint and weak staining of ROS1 IHC and other gene aberrance was not clear since not all faint and weak staining cases underwent EGFR mutation and ALK rearrangement detection. Future studies will include more patients carrying ROS1 arrangements (i.e. young, female, non-smokers with adenocarcinoma) to obtain more positive cases for the analysis.

## Conclusions

In conclusion, IHC can be a reliable and effective method for ROS1 rearrangements preliminary screening in patients with NSCLC thanks to the high sensitivity and specificity of IHC using the D4D6 mAb, while cutoff should be set at diffusely (≥60 % tumor cells) 2–3+ cytoplasmic reactivity and an H-score ≥150. FISH and qRT-PCR can be used as confirmation analysis. Young, female, and non-smoking patients with adenocarcinoma and without other RTKs aberrance should undergo tests for ROS1 rearrangement because patients with these characteristics may carry ROS1 rearrangement. TKI therapy with crizotinib is effective in patients with ROS1 rearrangements at advanced stages.

## Abbreviations

ADC, adenocarcinoma; ALK, anaplastic lymphoma receptor tyrosine kinase; ARMS-PCR, amplification refractory mutation system polymerase chain reaction; ASC, adenosquamous carcinoma; EGFR, epidermal growth factor receptor; FISH, fluorescent *in situ* hybridization; H&E staining, hematoxylin and eosin staining; IHC, immunohistochemistry; KRAS, Kirsten rat sarcoma viral oncogene homolog; LCLC, Large-cell lung carcinoma; mAb, monoclonal antibody; MET, MET proto-oncogene, receptor tyrosine kinase; NSCLC, non-small cell lung cancer; qRT-PCR, quantitative real-time polymerase chain reaction; ROS1, ROS proto-oncogene 1, receptor tyrosine kinase; RTKs, receptor tyrosine kinases; SCC, Squamous cell carcinoma; TKIs, tyrosine kinase inhibitors

## Additional files

Additional file 1: Table S1. The details of adenocarcinoma grading. (DOCX 64.6 kb) Additional file 2: Figure S1. Direct sequencing of the six cases with ROS1 rearrangement. (ZIP 4.08 mb) Additional file 3: Figure S2. The IHC staining patterns of ROS1 rearrangement cases and non-rearrangement cases. (ZIP 7.10 mb) Additional file 4: Figure S3. The details of follow-up studies. (ZIP 16.5 mb) Additional file 5: Public data. The dataset of the experiment. (XLS 86 kb)
