# Supplementary material for: Comparison of detection methods and follow-up study on the tyrosine kinase inhibitors therapy in non-small cell lung cancer patients with ROS1 fusion rearrangement
Source: BMC Cancer. 2016 Aug 4;16:599. doi: 10.1186/s12885-016-2582-9 (PMC4973062; doi:10.1186/s12885-016-2582-9)
Supplement: Additional file 1: Table S1. — The details of adenocarcinoma grading. (DOCX 64.6 kb) [file 12885_2016_2582_MOESM1_ESM.docx]

**Additional Table 1** Details of adenocarcinoma grading

| Grading method | Criteria | ROS1 rearrangement | ROS1 non-rearrangement |
| --- | --- | --- | --- |
| Sica grading |  |  |  |
| Score 2 | AIS or MIA^a^ | 0 | 6 |
| Score 3 | Mix subtype, well differentiate lepidic^b^, acinar or papillary pattern | 3 | 45 |
| Score 4 | Mix subtype, acinar, papillary or lepidic pattern with solid or micropapillary pattern; Pure acinar or pure papillary | 1 | 47 |
| Score 5 | Mix subtype, poorly differentiated with acinar/papillary and micropapillary or solid patterns | 4 | 65 |
| Score 6 | Mixed subtype, poorly differentiated with predominant solid and micropapillary patterns. Pure solid  or pure micropapillary | 0 | 10 |
| WHO grading |  |  |  |
| Low | lepidic adenocarcinoma | 1 | 16 |
| Intermediate | acinar or papillary adenocarcinoma | 5 | 121 |
| High | solid or micropapillary adenocarcinoma | 2 | 36 |

^a^The Sica grading was according to the 2004 WHO classification of lung cancer, which used the term bronchioloalveolar carcinoma (BAC) to describe noninvasive component. In the 2015 WHO classification, the term has been replaced by adenocarcinoma *in situ* (AIS) and minimally invasive adenocarcinoma (MIA)

^b^The term “well differentiated BAC” is equal to the term lepidic in the 2015 WHO classification
