# Supplementary material for: Comparison of detection methods and follow-up study on the tyrosine kinase inhibitors therapy in non-small cell lung cancer patients with ROS1 fusion rearrangement
Source: BMC Cancer. 2016 Aug 4;16:599. doi: 10.1186/s12885-016-2582-9 (PMC4973062; doi:10.1186/s12885-016-2582-9)
Supplement: Additional file 2: Figure S1. — Direct sequencing of the six cases with ROS1 rearrangement. (ZIP 4.08 mb) [file 12885_2016_2582_MOESM2_ESM.zip › Legend of Additional Fig1.docx]

**Additional Fig. 1** Direct sequencing of the six cases with ROS1 rearrangement

Six cases with ROS1 rearrangement, which were detected by IHC, FISH and qRT-PCR had undergone direct sequencing to reveal the fusion types. The order of the cases is according to the order in Table.2. The red marks in the figure are pointing out the fusion sites. (a–1) & (a–2) Case 1 was harboring two fusion types, which were SLC34A2-E4, ROS1-E32 and SLC34A2-E4, ROS1-E34, respectively; (b) The fusion type of case 2 was CD74-E6, ROS-E34; (c) Case 3 was also harboring CD74-E6, ROS-E34 rearrangement; (d) The fusion type of case 4 was TPM3-E8, ROS1-E35; (e–1) & (e–2) Case 5 was harboring two fusion types, which were SLC34A2-E14del, ROS1-E32 and SLC34A2-E14del, ROS1-E34, respectively; (f) The fusion type of case 6 was CD74-E6, ROS-E34
