# Supplementary figures and images for: Comparison of detection methods and follow-up study on the tyrosine kinase inhibitors therapy in non-small cell lung cancer patients with ROS1 fusion rearrangement
Source: BMC Cancer. 2016 Aug 4;16:599. doi: 10.1186/s12885-016-2582-9 (PMC4973062; doi:10.1186/s12885-016-2582-9)

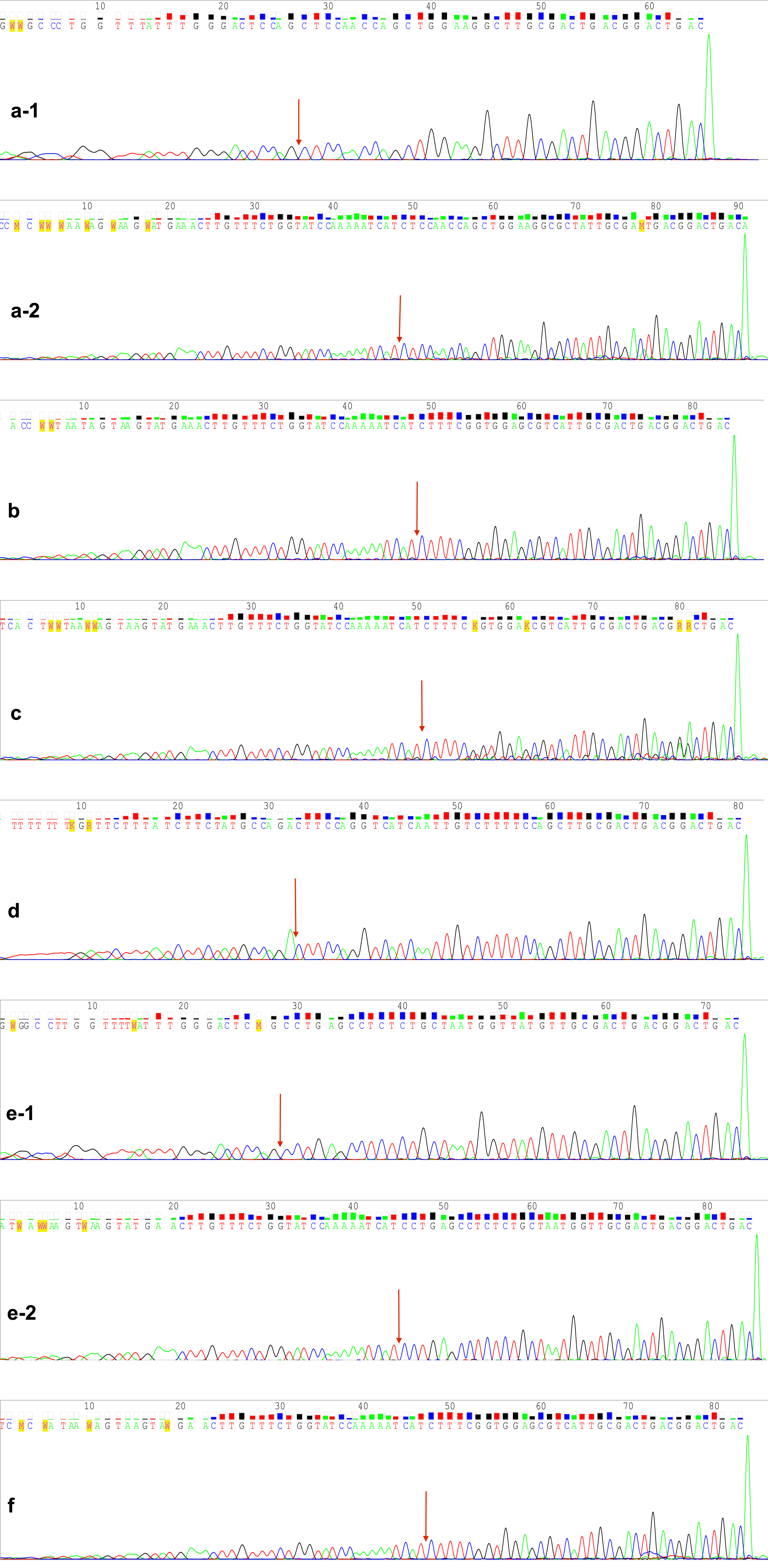

Supplement: Additional file 2: Figure S1. — Direct sequencing of the six cases with ROS1 rearrangement. (ZIP 4.08 mb) [file 12885_2016_2582_MOESM2_ESM.zip › Additional Fig1(5.26).tif]

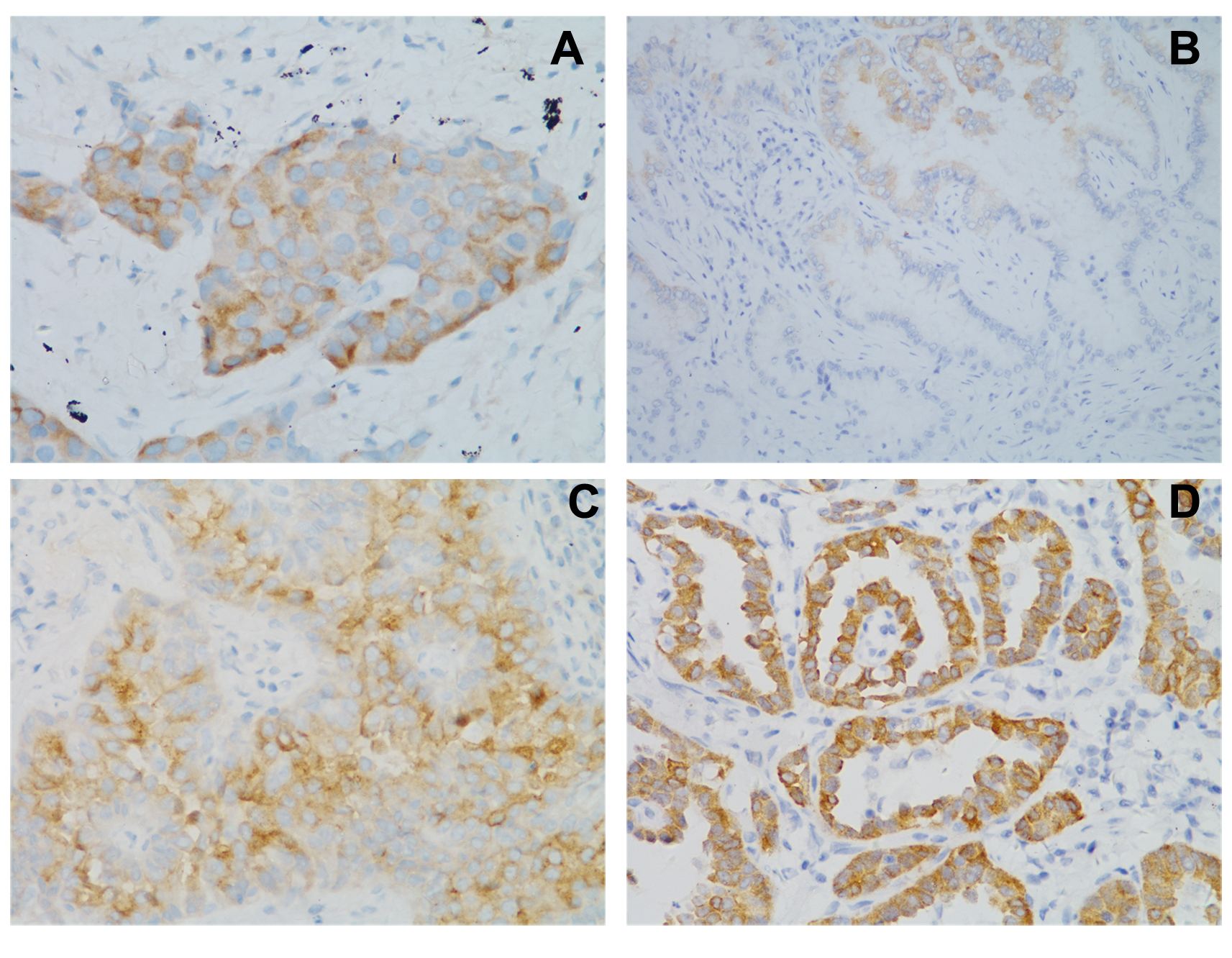

Supplement: Additional file 3: Figure S2. — The IHC staining patterns of ROS1 rearrangement cases and non-rearrangement cases. (ZIP 7.10 mb) [file 12885_2016_2582_MOESM3_ESM.zip › Additional Fig2.tif]

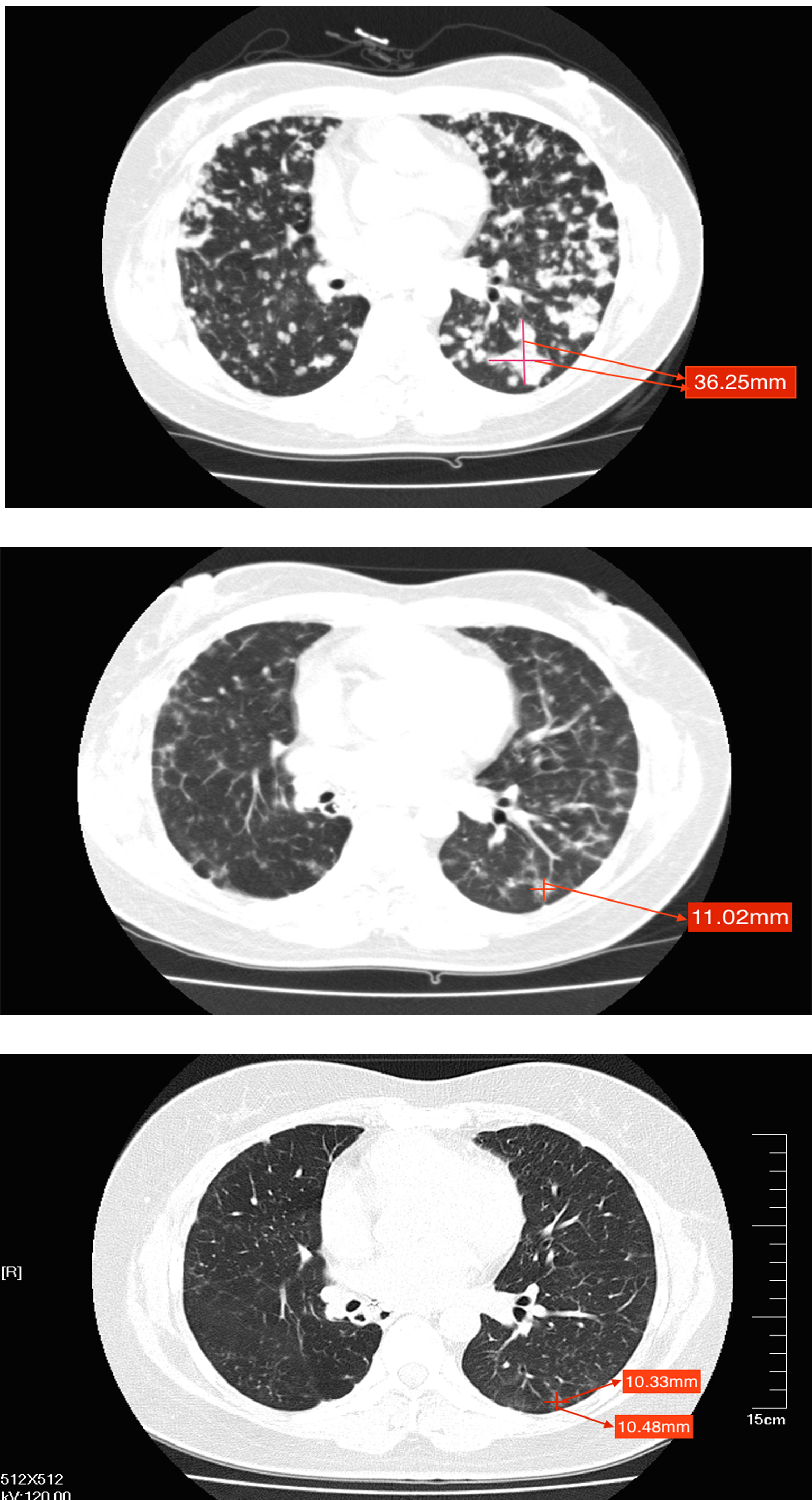

Supplement: Additional file 4: Figure S3. — The details of follow-up studies. (ZIP 16.5 mb) [file 12885_2016_2582_MOESM4_ESM.zip › Additional Fig3-b.tif]

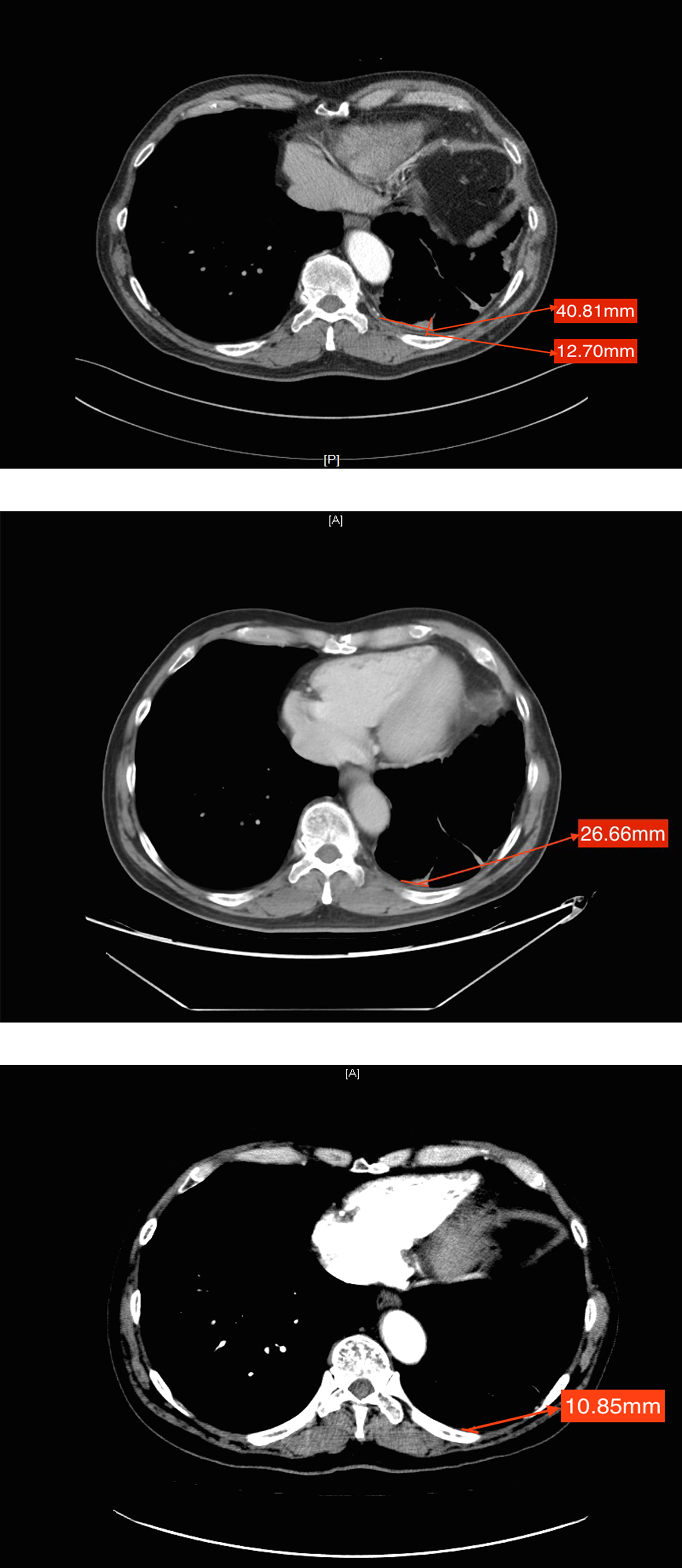

Supplement: Additional file 4: Figure S3. — The details of follow-up studies. (ZIP 16.5 mb) [file 12885_2016_2582_MOESM4_ESM.zip › Additional Fig3-a.tif]

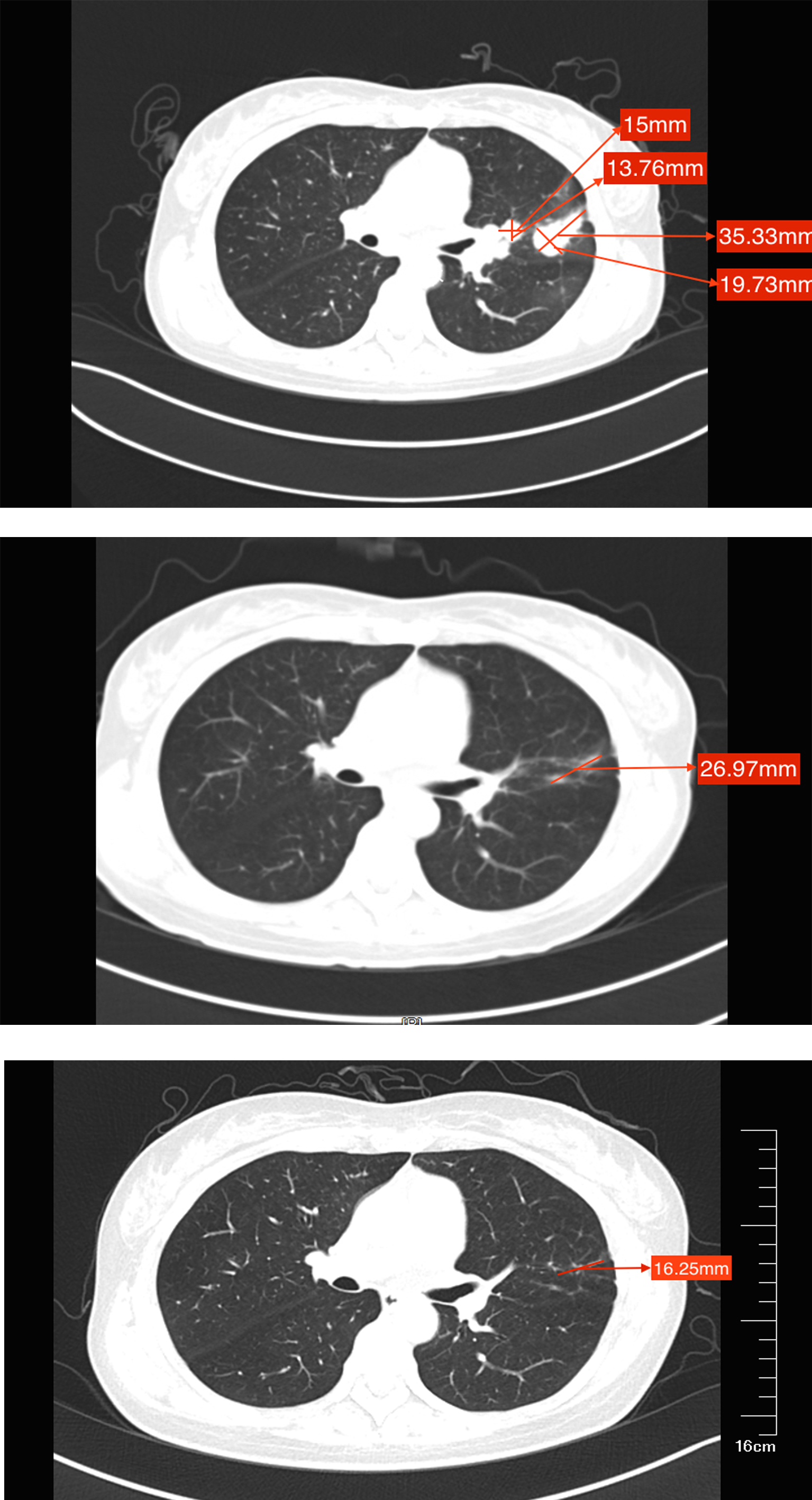

Supplement: Additional file 4: Figure S3. — The details of follow-up studies. (ZIP 16.5 mb) [file 12885_2016_2582_MOESM4_ESM.zip › Additional Fig3-c.tif]
