# Supplementary material for: Comparison of detection methods and follow-up study on the tyrosine kinase inhibitors therapy in non-small cell lung cancer patients with ROS1 fusion rearrangement
Source: BMC Cancer. 2016 Aug 4;16:599. doi: 10.1186/s12885-016-2582-9 (PMC4973062; doi:10.1186/s12885-016-2582-9)
Supplement: Additional file 3: Figure S2. — The IHC staining patterns of ROS1 rearrangement cases and non-rearrangement cases. (ZIP 7.10 mb) [file 12885_2016_2582_MOESM3_ESM.zip › Legend of Additional Fig2.docx]

**Additional Fig. 2** IHC staining patterns of ROS1 rearrangement cases and non-rearrangement cases

(a) A case showed moderate staining (2+) in ROS1 IHC with focal distribution (about 40 % tumor cells) and an H-score of 90, which was proved as ROS1 non-rearrangement by FISH (400×); (b) Another case with an H-score of 10, showing weak (1+) and focal staining (about 10 % tumor cells) of ROS1 IHC. The staining mainly present adjacent to the lumen in the tumor cells, and the case was also proved as ROS1 non-rearrangement by FISH (200×); (c) A case has been proved as ROS1 rearrangement by FISH and qRT-PCR, showing diffusely (about 80 % tumor cells) moderate staining (2+) in IHC with cytoplasmic and focal granular staining pattern, and an H-score of 180 (400×); (d) Another case showed diffusely (about 90 % tumor cells) strong staining (3+) in ROS1 IHC with cytoplasmic and focal granular staining patter, and an H-score of 250. It was also verified as ROS1 rearrangement by FISH (400×)
