# Supplementary material for: Comparison of detection methods and follow-up study on the tyrosine kinase inhibitors therapy in non-small cell lung cancer patients with ROS1 fusion rearrangement
Source: BMC Cancer. 2016 Aug 4;16:599. doi: 10.1186/s12885-016-2582-9 (PMC4973062; doi:10.1186/s12885-016-2582-9)
Supplement: Additional file 4: Figure S3. — The details of follow-up studies. (ZIP 16.5 mb) [file 12885_2016_2582_MOESM4_ESM.zip › Additional file3(5.25).docx]

**Additional file. 3** The details of follow-up studies

Patient 3 has a history of video-assisted thoracic surgery (VATS) of the left upper lobe wedge resection. The histopathology indicated invasive mucinous adenocarcinoma with pleural invasion. The patient had received chemotherapy with pemetrexed and carboplatin after operation. Later, CT image revealed shadows in left thoracic wall, and the largest lesion was approximate in size to 40.81 × 12.70 mm^2^. The patient was proved as ROS1 rearrangement by IHC, FISH and qRT-PCR detection, and confirmed by direct sequencing as CD74-E6 fusion rearrangement. Finally, the patient received crizotinib therapy with 250 mg bid from December 2014. Two months later, the CT image showed the lesion in the thoracic wall had decreased to 26.66 × 11.69 mm^2^ in size, indicating the patient was in partial response (PR) (by RECIST guideline 1.1 [18]). In October 2015, CT image showed the lesion shrunk to 10.85 × 8.60 mm^2^ in size. The major complain were tiredness and constipation during therapy. The CT images during therapy were showed in an additional figure [see Additional Fig 3, a].

Patient 6 has a history of lower right lobe radical resection. Histopathology indicated invasive adenocarcinoma with acinar predominant subtype. Five years later, the disease relapsed with metastasis, and the CT image indicated the largest lesion was approximated 36.25 × 36.25 mm^2^ in size on the pleura. After chemotherapy of pematrexed, nadaplatin and bevacizumab, biopsy had taken from the patient’s pleura, and the histopathology revealed invasive adenocarcinoma with ROS1 rearrangement (by IHC, FISH and qRT-PCR detection). Direct sequencing revealed that the fusion type was CD74-E6. The patient was subsequently undergoing crizotinib (250 mg bid) therapy from April 2014. Ten days later, the CT image showed the largest lesion decrease to 11.02 × 8.59 mm^2^ in size, indicating the patient turned to PR. In October 2015, CT image showed the largest lesion shrunk to 10.48 × 10.33 mm^2^ in size. The major complain were edema in lower limbs, vomiting and tiredness during therapy. Cardiac disorder was excluded. The CT images during therapy were showed in an additional figure [see Additional Fig 3, b].

Patient 7 took CT scan during health examination, suggesting lung cancer with metastasis. The largest lesion was approximate in size to 35.33 × 19.73 mm^2^. A biopsy and subsequent histopathological analysis revealed invasive adenocarcinoma with ROS1 arrangement (by FISH and IHC detection). Therefore, the patient was given crizotinib (250 mg bid) from July 2014. After two months of therapy, CT image showed that the largest foci in left lung had decreased 26.97 × 15.12 mm^2^ in size, and that the lesion in the hilum had disappeared, indicating that this patient had undergone PR. In November 2015, the largest lesion shrunk to 16.25 × 5.65 mm^2^ in size. The major complain of the patient was tiredness during therapy. The CT images during therapy were showed in an additional figure [see Additional Fig 3, c].
